# Impact of JAK2 V617F Mutation on Hemogram Variation in Patients with Non-Reactive Elevated Platelet Counts

**DOI:** 10.1371/journal.pone.0057856

**Published:** 2013-02-28

**Authors:** Juan Zhou, Yuanxin Ye, Shugen Zeng, Yi Zhou, Zhigang Mao, Xingbo Song, Binwu Ying, Xiaojun Lu, Hong Jiang, Lanlan Wang

**Affiliations:** Department of Laboratory Medicine, West China Hospital, Sichuan University, Chengdu, Sichuan Province, China; University of Leuven, Belgium

## Abstract

**Background:**

Non-reactive platelet counts elevation occurs mainly in myeloproliferative disorders (MPDs), which have been reported to be closely associated with JAK2 V617F mutation. Complete blood cell count (CBC) is essential in diagnosis of MPDs, however, the impact of JAK2 V617F mutation on the patients’ hemogram variation remains not clear.

**Methods:**

JAK2 V617F mutation was detected by allele specific real-time quantitative fluorescence PCR (AS-qPCR).

**Results:**

Of the 402 non-reactive platelet elevating patients, JAK2 V617F mutation was detected in 222 (55.2%) patients. RBC counts, WBC counts, platelet-large contrast ratio (P-LCR), platelet distribution width (PDW) and mean platelet volume (MPV) were much higher in JAK2 V617F mutated patients, except platelet counts. In addition, when the patients were classified into subgroups by blood cell counts, it was found that JAK2 V617F mutation rate increased progressively with the increase of RBC counts and WBC counts, other than platelet counts. Furthermore, trilineage hyperplasia group showed highest JAK2 V617F mutation rate (93.26%), followed by the bilineage hyperplasia groups. Lastly, JAK2 V617F mutant allele burden was found much higher in polycythemia vera (PV) patients [median(P_25_–P_75_): 45.02%(35.12%–54.22%)] than in essential thrombocythemia (ET) patients [median(P_25_–P_75_): 28.23%(17.77%–41.66%)], and that it increased with WBC counts (r = 0.393, p = 0.000) and RBC counts(r = 0.215, p = 0.001), other than platelet counts (r = −0.051, p = 0.452). Further analysis revealed that in ET patients, JAK2 V617F mutant allele burden correlated with WBC counts and platelet counts positively, other than RBC counts, while in PV patients, it correlated with WBC counts and RBC counts positively, but not platelet counts.

**Conclusions:**

JAK2 V617F mutation occurs frequently in patients with non-reactive elevated platelet counts. The presence of JAK2 V617F mutation has great impact on hemogram variation, including RBC counts, WBC counts, platelet parameters and lineage hyperplasia, but not on platelet counts. Besides, JAK2 V617F mutant allele burden affects the blood cell proliferation pattern.

## Introduction

Platelet is essential to initiate thrombosis following vascular injury and repair the minute vascular damage that continuously occurs [1]. Platelet count in normal people ranges from 100 to 300×10^9^/L in Chinese population. Excessive platelet may result in vascular complications, such as thrombosis, microvascular disturbances and hemorrhage [2]. Elevated platelet count is primarily found in two conditions. One condition is “reactive thrombocytosis”, which is usually due to an external cause, such as chronic inflammation, cancer, iron deficiency, or following splenectomy, and the platelet elevating could be corrected by curing the primary disease. The other is “non-reactive”, caused by alterations targeting the hematopoietic cells [3], occurs mostly in myeloproliferative disorders (MPDs), which are characterized by multipotent hematopoitic stem cell derived clonal myeloproliferation, resulting in increased blood cell production related to cytokine hypersensitivity and virtually normal cell maturation [4]. The second condition requires special treatment, such as myelosuppressive drugs [5] and JAK inhibitors [6]–[7].

An acquired mutation in the Janus kinase 2 (JAK2) gene was discovered in a high proportion of patients with MPDs in 2005 [8]. This mutation is a base replacement (1849G>T) in the Jak homology domain 2(JH2) of JAK2 and leads to a valine to phenylalanine substitution at codon 617(V617F or JAK2 V617F mutation) [8]–[11]. There are three major types of MPDs: polycythemia vera (PV), essential thrombocythemia (ET) and primary myelofibrosis(PMF). JAK2 V617F mutation was observed in almost all PV patients, approximately half ET and PMF patients, but rarely in patients with CML and atypical MPDs [12]–[14]. The above mentioned diseases, especially ET and PV, account for the majority of patients with non-reactive elevated platelet counts, suggesting that JAK2 V617F plays an important role in patients with non-reactive elevated platelet counts.

It has been demonstrated that JAK2 V617F mutation plays a direct causative role in MPD pathogenesis [15]. JH2 is crucial for inhibition of basal Jak activity [16], and JAK2 V617F mutation in this domain abolishes its autoinhibition, thereby results in persistent activation of JAK/STAT pathway, which usually activated by hematopoietic growth factors through specific receptors [15]. JAK2 V617F increases Bcl-xL expression in hematopoietic progenitor cells [17] and contributes to the development of erythrocytosis [18]. In addition, JAK2 V617F induces abnormal activation of thrombopoietin receptor (TPOR) and granulocyte colony-stimulating factor receptor (G-CSFR) pathway, leading to hyperplasia of megakaryocytic and granulocytic progenitor cells [19]. Because JAK2 V617F mutation promotes proliferation of multiple cell lineages, patients with such mutation exhibit increased numbers of mature and immature cells in peripheral blood.

Several studies have showed that hemogram is altered in patients with JAK2 V617F mutation. In a study of 806 ET patients at diagnosis [20], hemoglobin (Hb), white blood cell (WBC) counts and neutrophil counts were found higher in patients with JAK2 V617F than patients without JAK2 V617F, while platelet showed the opposite correlation. In another study for ET patients [21], no significant difference was found for WBC counts in the mutated and wild-type groups. However, Ana L. Basquiera et al [22] reported that WBC counts and Hb were significantly higher in JAK2 V617F mutated group than that in wild-type group in ET and PV patients, but platelet counts showed no significant difference. Therefore, the relationship between JAK2 V617F and hemogram variation was controversial, and no investigation has been performed to determine the JAK2 V617F mutation rate in patients groups classified by RBC counts, WBC counts, platelet counts and lineage hyperplasia state. Besides, when considering platelet parameters, most studies focused on thrombopenia, there has been only one study [23] exploring the relationship between JAK2 V617F mutation and platelet parameters in ET patients, including platelet distribution width (PDW) and mean platelet volume (MPV).

JAK2 V617F allele burden might affect phenotypes of MPD, because low JAK2 V617F allele burden in hematopoietic cells was associated with a phenotype resembling ET, whereas a higher burden was associated with a phenotype resembling PV [24]–[25]. It was suggested that megakaryopoiesis is strongly enhanced while erythropoiesis is only slightly stimulated when the progenitors are heterozygous for JAK2 V617F, and in contrast, erythropoiesis is strongly stimulated when the progenitors are homozygous for JAK2V617 [3]. Therefore, we hypothesis that JAK2 V617F mutant allele burden may also influence blood cell proliferation pattern.

Since JAK2 V617F plays an important role in patients with non-reactive elevated platelet counts and greatly influences hyperplasia of several cell lineages, the current study was aiming to investigate the relationship between JAK2 V617F mutation and hemogram variation of non-reactive platelet counts elevating patients in details.

## Materials and Methods

### Study Population

A retrospective study was conducted for patients visiting our hospital (West China Hospital of Sichuan University) from April 2009 to September 2012. A total of 402 unrelated patients with elevated platelet count but negative for BCR-ABL gene were included. Of note, BCR-ABL gene was determined to exclude typical chronic myelocytic leukemia, which is independent of JAK2 V617F mutation. Platelet count elevation was defined as platelet counts >300×10^9^/L, according to the platelet count reference range in China, and the increase in platelet counts was non-reactive by excluding external causes, such as chronic inflammation, cancer, iron deficiency, or following splenectomy [3].

### Ethics Statement

Written Informed consents were obtained from all included individuals and this study was approved by the ethical committee of West China Hospital, Sichuan University.

### JAK2 V617F Mutation Detection

Total DNA was extracted from EDTA-anticoagulated peripheral blood or bone marrow samples by using QIAGen DNA Blood Mini kits (QIAGEN, Hilden, Germany) following the manufacturer’ s instructions. Allele specific real-time quantitative fluorescence PCR (AS-qPCR) was performed to detect JAK2 V617F mutation using a quantitative fluorescent DNA detection kit (Shenyou, shanghai, China). JAK2 wild type and mutation type were both amplified with respective primers, and JAK2 V617F mutant allele burden was relatively quantified (JAK2 V617F %) by determining the percentage of JAK2 mutation type quantity in the total JAK2 quantity.

### Blood Routine Examination

Blood routine examination including red blood cell (RBC) counts, white blood cell (WBC) counts, platelet counts and corresponding platelet parameters was examined using the fully automated hematology analyzer XE-5000™ (Sysmex, Kobe, Japan).

### Bone Marrow Smear Cytology

The bone marrow smears were stained with Wright's-Giemsa, and examined by light microscopy. Two hundred nucleated cells were classified and the percentages of all types of cells were calculated. BM granulopoiesis (%) and BM erythropoiesis(%) represented the percentage of granulocytes and nucleated erythrocytes, respectively. Granulopoiesis/erythropoiesis was the ratio of granulocytes to nucleated erythrocytes.

### Statistical Analysis

The patients were categorized according to their red blood cell (RBC) counts, white blood cell (WBC) counts and platelet counts in the peripheral blood, and the frequencies of JAK2 V617F mutation in the groups were calculated and compared using chi-square and Fisher’s exact probability test. The relative quantity of JAK2 V617F mutation burden was presented as median (P_25_–P_75_), and Kruskal-Wallis test was used to compare the differences among groups. The age difference between JAK2 V617F mutated and wild-type groups was compared using t test. Comparison of RBC count, WBC count, platelet count and platelet parameters between JAK2 V617F mutated and wild-type groups were performed using Kruskal-Wallis test. Correlation analysis between JAK2 V617F mutant allele burden and blood cell counts, bone marrow findings were performed using spearman correlation test. All analyses were performed using SPSS 17.0. Probability values less than 0.05 were considered to be statistically significant. When comparing the mutation rate in two of the four groups, we used an adjusted α = 0.0042(α′ = α/[k(k−1)], k: number of groups) as the significant level.

## Results

### Basic Characteristics of Studied Patients

Among 402 patients enrolled in the current study with non-reactive elevated platelet counts, over half (222, 55.2%) of them carried JAK2 V617F mutation. The average age of the patients was 53.3 years, and M/F was 185/217. Since most of the patients with non-reactive elevated platelet count were ET and PV patients, we further investigated the relationship between JAK2 V167F mutation and blood cell counts in 185 ET and 57 PV patients, respectively, diagnosed strictly according to WHO diagnostic criteria. The JAK2 V617F mutation rates in ET and PV patients were 57.8% (107/185) and 96.5% (55/57), respectively. Of note, we found that patients with JAK2 V617F mutation were older than those without mutation. However, gender seemed to be irrelative with mutation state, as shown in Table 1.

**Table 1:** Basic characteristic of the patients and blood cell counts in JAK2 V617F mutation positive and negative patients.

		JAK2 V617F mutation		
	All patients	negative	Positive	P
Number (%)				
All patients	402	180(44.8%)	222(55.2%)	
ET patients	185	78(42.2%)	107(57.8%)	
PV patients	57	2(3.5%)	55(96.5%)	
Age(years), mean±SD				
All patients	53.3±18.0	47.6±18.5	57.9±16.2	0.000
ET patients	52.3±18.7	47.1±18.2	56.1±18.2	0.001
PV patients	60.7±11.4	52.0±14.1	61.0±11.3	/*
Gender(Male/Female)				
All patients	185/217	75/105	110/112	0.115
ET patients	82/103	32/46	50/57	0.441
PV patients	31/26	0/2	31/24	/*
RBC count(×10^12^/L), median(P_25_–P_75_)				
Male				
All patients	4.83(4.03–5.63)	4.34(3.41–4.85)	5.32(4.34–6.59)	0.000
ET patients	4.84(4.25–5.27)	4.74(4.28–5.04)	5.02(4.13–5.39)	0.109
Female				
All patients	4.35(3.96–5.11)	4.12(3.70–4.40)	4.89(4.20–5.80)	0.000
ET patients	4.46(4.09–4.96)	4.16(3.81–4.47)	4.76(4.29–5.16)	0.000
Platelet count(×10^9^/L), median(P_25_–P_75_)				
All patients	672(474–975)	679(503–969)	672(455–994)	0.775
ET patients	901(688–1233)	834(637–1107)	932(715–1381)	0.077
WBC count(×10^9^/L), median(P_25_–P_75_)				
All patients	11.11(7.80–15.94)	8.66(6.70–12.35)	13.25(9.99–20.65)	0.000
ET patients	10.38(8.15–14.59)	8.59(6.82–10.67)	12.37(9.85–18.16)	0.000

* Comparison in PV patients is not performed due to the small sample size of JAK2 V617F mutation negative patients (n = 2).

### Relationship between JAK2 V617F Mutation and Blood Cell Counts

The medians of RBC counts were 4.83×10^12^/L for males and 4.35×10^12^/L for females. The median of RBC counts of mutated patients was higher than that of wild-type patients in both males (5.32×10^12^/L *vs* 4.34×10^12^/L) and females (4.89×10^12^/L *vs* 4.12×10^12^/L). The median of WBC counts was also much higher in JAK2 V617F mutated patients(13.25×10^9^/L) than that in wild-type patients(8.66×10^9^/L). In contrast, no significant difference was found when comparing platelet count. As shown in Table 1, similar phenomenon was also observed in ET patients, while platelet counts were slightly higher in JAK2 mutated patients, although not reached a significant level. Comparison of blood cell counts between JAK2 V617F mutated and wild-type PV patients was not performed, due to small sample size of the JAK2 V617F mutated PV patients(n = 2).

Furthermore, we determined BM granulopoiesis (%), BM erythropoiesis (%), Granulopoiesis/erythropoiesis in the bone marrow for both JAK2 V617F mutated and wild-type patients. No significant difference was detected either in all of the patients or in ET patients (data not shown), suggesting equal impact of JAK2 mutation on granulopoiesis and erythropoiesis in the bone marrow.

### JAK2 V617F Mutation Rates in Patient Groups Classified According to Blood Cell Counts and Lineage Hyperplasia State

To further investigate relationship between JAK2 V617F mutation and blood cell counts, we divided patients according to classification of RBC counts, WBC counts and platelet counts, based on the reference range and diagnosis criteria of MPDs (Table 2).

**Table 2:** JAK2 V617F mutation rates of different groups of patients classified by blood cell counts and linage hyperplasia state.

	All patients			ET patients		
	N	JAK2 V617F positive (n/%)	P	N	JAK2 V617F positive (n/%)	P
RBC count (×10**^12^/L**)						
<4.0(<3.5)*	70	26(37.14%)	0.000	21	10(47.62%)	0.000
4.0–5.5(3.5–5.0)*	223	98(43.95%)		130	66(50.77%)	
5.5–6.5(5.0–6.0)*	50	41(82.00%)		31	28(90.32%)	
>6.5(>6.0)*	59	57(96.61%)		3	3(100.00%)	
WBC count (×10**^9^/L**)						
<4.0	11	1(9.09%)	0.000	2	0(0.00%)	0.000
4.0–10.0	162	55(33.95%)		85	30(35.29%)	
10.0–50.0	224	162(72.32%)		97	76(78.35%)	
>50.0	5	4(80%)		0	/	
Platelet count (×10**^9^/L**)						
300–450	83	52(62.65%)	0.211	0	/	0.266
450–600	84	40(47.62%)		29	12(41.38%)	
600–1000	143	76(53.15%)		80	48(60.00%)	
>1000	92	54(58.70%)		76	47(61.84%)	
Hyperplasia						
Isolated PLT↑	153	41(26.80%)	0.000	79	24(30.38%)	0.000
PLT+WBC ↑	140	83(59.29%)		8	6(75.00%)	
PLT+RBC ↑	20	15(75.00%)		72	52(72.22%)	
PLT+WBC+RBC↑	89	83(93.26%)		26	25(96.15%)	

* The number outside the parentheses presented RBC count of males, while the number inside the parentheses presented RBC count of females, owing to different reference range of RBC count in males and females.

**Figure 1:**
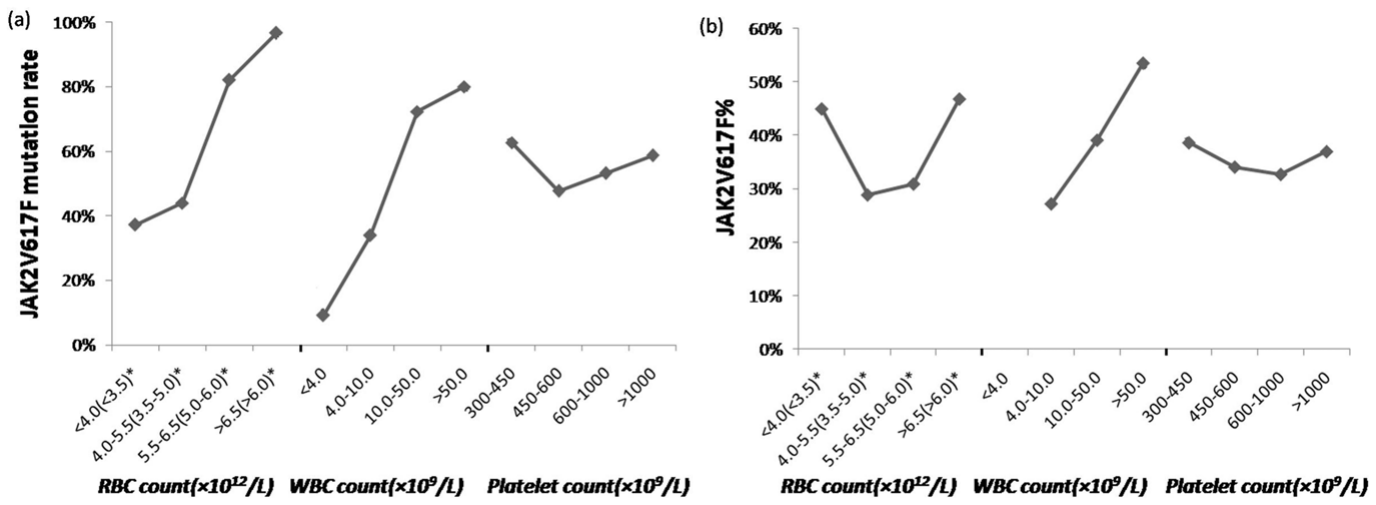
Trends of JAK2 V617F mutation rate (a) and JAK2 V617F mutant allele burden (b) variation in groups classified by blood cell counts. *The number outside the parentheses presented RBC count of males, while the number inside the parentheses presented RBC count of females, owing to different reference range of RBC count in males and females. The vertical axis represents JAK2 V617F mutation rate (a) and the median of JAK2 V617F mutant allele burden (b) in groups classified by blood cell counts. Median of JAK2 V617F mutant allele burden in the group which was characterized by WBC counts <4.0×10^9^/L was not shown in the figure, since only one patient was included into this group.

Patients were categorized into four groups by RBC counts (×10^12^/L): <4.0(<3.5), 4.0–5.5(3.5–5.0), 5.5–6.5(5.0–6.0), >6.5(>6.0). Owing to the different reference range of RBC count in males and females, RBC count of males and females were presented separately, the former is outside the parentheses and the latter is inside the parentheses in Table 2. JAK2 V617F mutation rates in the four groups were 37.14%, 43.95%, 82.00%, 96.61%, respectively, and the rates showed significant difference assessed by chi-square test. It was found that the p values were close to zero between any two neighboring groups, suggesting that JAK2 V617F mutation rate was increasing progressively with the increase of RBC count, as shown in Figure 1a. Further analysis was performed in ET patients, and a similar trend was observed, as shown in Table 2.

When we divided patients into four groups according to WBC counts (×10^9^/L): <4.0, 4.0–10.0, 10.0–50.0, >50.0, similar results were obtained as we observed in the analyses for classification with RBC counts. JAK2 V617F mutation rate in the four groups were 9.09%, 33.95%, 72.32%, 80.00%, respectively, and the rates were significant different assessed using chi-square test. It was found that the p values were close to zero between any two neighboring groups, suggesting that JAK2 V617F mutation rate was also increasing progressively with increase of WBC count, as shown in Figure 1a. Of note, WBC counts of most ET patients were in the range of (4.0–50.0)×10^9^/L, the mutation rates of the groups with WBC counts ranged (4.0–10.0)×10^9^/L and (10.0–50.0)×10^9^/L were 35.29% and 78.35%, respectively.

However, when considering platelet count classification, no significant difference was found among the four groups. The mutation rates of the four groups were all around 50%. Similarly, JAK2 mutation rates did not show significant difference among the four groups in ET patients either. However, we could find that JAK2 mutation rates in the patients with platelet counts ranged 600–1000×10^9^/L (60.00%) and >1000×10^9^/L (61.84%) were higher than that in the patients with platelet counts ranged 450–600×10^9^/L (41.38%), although it did not reach a significant level.

It is well known that JAK2 mutation occurred in myeloproliferative diseases, such as polycythemia vera (PV), essential thrombocythemia (ET) and primary myelofibrosis (PMF). These diseases were characterized by monolineage, bilineage or trilineage hyperplasia. Therefore, in our further analyses, patients were classified into four groups according to lineage hyperplasia state: Isolated PLT↑, PLT+WBC↑, PLT+RBC↑, and PLT+WBC+RBC↑. The greatest difference was found in the four groups (p = 0.000). The highest mutation rate was found in PLT, WBC and RBC trilineage hyperplasia group (93.26%), followed by bilineage hyperplasia groups: PLT+WBC↑(59.29%) and PLT+RBC↑(75.00%), the lowest mutation rate was found in isolated PLT↑ group (26.80%), and comparisons between any two of the four groups all reached statistical significance even after adjusting for significant level. In addition, similar trends were found in ET patients, as shown in Table 2.

### Relationship between JAK2 V617F Mutation and Platelet Parameters

Platelet parameters including thrombocytocrit (PCT), platelet-large contrast ratio (P-LCR), platelet distribution width (PDW) and mean platelet volume (MPV) were compared between JAK2 V617F mutated and wild-type groups in 281 patients whose platelet parameters were available, and found that the medians of P-LCR, PDW, and MPV in mutated patients were higher than those in wild-type patients, while PCT was almost the same in the two groups. Furthermore, analysis was also performed in 134 ET patients and the silimar results were obtained (Table 3).

**Table 3:** Platelet parameters in JAK2 V617F mutation positive and negative patients.

		JAK2 V617F mutation		
	All patients	Negative	Positive	P
PCT(L/L), median(P_25_–P_75_)				
All patients	0.75(0.54–1.04)	0.75(0.52–1.00)	0.76(0.55–1.09)	0.413
ET patients	0.95(0.72–1.26)	0.93(0.72–1.22)	0.97(0.72–1.32)	0.481
P-LCR(%), median(P_25_–P_75_)				
All patients	29.2(23.1–35.9)	25.7(20.5–32.4)	32.2(26.2–36.9)	0.000
ET patients	29.1(23.5–35.5)	25.7(21.1–32.3)	30.8(25.9–36.1)	0.004
MPV(fL), median(P_25_–P_75_)				
All patients	10.5(9.8–11.3)	10.1(9.5–11.0)	10.9(10.1–11.4)	0.000
ET patients	10.5(9.9–11.3)	10.1(9.5–11.0)	10.7(10.1–11.4)	0.009
PDW(fL), median(P_25_–P_75_)				
All patients	12.6(11.1–14.4)	11.5(10.4–13.3)	13.3(11.8–15.0)	0.000
ET patients	12.4 (11.1–14.2)	11.4(10.4–13.5)	12.9(11.5–14.6)	0.001

PCT: thrombocytocrit; P-LCR: platelet-large contrast ratio; PDW: platelet distribution width; MPV: mean platelet volume.

### Correlation of JAK2 V617F Mutant Allele Burden with Hemogram Variation

To investigate relationship between JAK2 V617F mutant allele burden and blood cell hyperplasia state in the 222 patients who were JAK2 V617F mutated, Spearman rank correlation test was performed, and it was found that JAK2 V617F allele burden correlated with RBC counts (r = 0.215, p = 0.001) and WBC counts (r = 0.393, p = 0.000), whereas not with platelet counts (r = −0.051, p = 0.452). In the mean time, the medians of JAK2V167F allele burden were significantly different among subgroups classified by RBC counts, WBC counts, but similar among subgroups classified by platelet counts. JAK2 V617F allele burden increased with WBC counts, while it exhibited complicated variation in RBC counts analysis, as shown in Figure 1b.

It has been reported that JAK2 V617F mutant allele burden contribute to different phenotypes of MPDs [26]. Accordingly, we compared the JAK2 V617F allele burden between 107 ET and 55 PV patients with JAK2 V617F mutation and found that JAK2 V617F allele burden was much higher in PV patients[median(P_25_–P_75_): 45.02% (35.12%–54.22%)] than in ET patients [median(P_25_–P_75_): 28.23%(17.77%–41.66%)]. Furthermore, Spearman rank correlation test was performed to assess the relationship between JAK2 V617F mutant allele burden and blood cells hyperplasia state in ET and PV patients separately. In ET patients, JAK2 V617F allele burden was correlated with platelet counts (r = 0.369, p = 0.000) and WBC counts (r = 0.424, p = 0.000), other than RBC counts (r = 0.061, p = 0.530); In contrast, JAK2 V617F allele burden in PV patients was correlated with WBC counts (r = 0.361, p = 0.007) and RBC counts (r = 0.330, p = 0.014), other than PLT counts (r = 0.111, p = 0.418). The results were shown in Table 4. Lastly, we investigated the impact of JAK2 V617F allele burden on bone marrow hyperplasia, and found that BM granulopoiesis (%) increased with JAK2 V617F allele burden, other than BM erythropoiesis (%) in ET patients, but opposite results were obtained in PV patients, although not to a significant extent, as shown in Table 4. These data, suggested that BM granulopoiesis was greater than erythropoiesis in ET patients, but was opposite in PV patients, which was consistant with their peripheral blood cell variations.

**Table 4:** Spearman’s rank correlation between JAK2 V617F allele burden and peripheral blood cell counts, bone marrow hyperplasia.

	JAK2V61F allele burden					
	All patients		ET patients		PV patients	
	R*	P	R*	P	R*	P
**Peripheral blood cell counts**						
RBC counts	0.215	0.001	0.061	0.530	0.330	0.014
WBC counts	0.393	0.000	0.424	0.000	0.361	0.007
PLT counts	−0.051	0.452	0.369	0.000	0.111	0.418
**Bone marrow hyperplasia**						
BM granulopoiesis (%)	0.070	0.325	0.250	0.011	−0.099	0.497
BM erythropoiesis (%)	0.053	0.456	−0.130	0.192	0.233	0.107
Granulopoiesis/erythropoiesis	−0.014	0.840	0.175	0.077	−0.197	0.175

* R: Spearman’s rank correlation coefficient.

## Discussion

In the present study, we investigated relationship between JAK2 mutation (JAK2 V617F) and hemogram variation in 402 BCR-ABL negative patients with non-reactive elevated platelet counts. Our analyses provide important information to understand the role of JAK2 V617F in influencing hemogram. First, JAK2 V617F mutation was detected in 55.2% of the enrolled patients, and JAK2 V617F mutation rate was related with lineage hyperplasia state, increased with RBC counts and WBC counts but not platelet counts. In addition, Platelet-large contrast ratio (P-LCR), platelet distribution width (PDW), and mean platelet volume (MPV) in JAK2 V617F-mutated patients were higher than those in wild-type patients. Furthermore, JAK2 V617F allele burden was correlated with RBC counts and WBC counts, but not PLT counts. Specifically, JAK2 V617F allele burden was correlated with WBC counts and PLT counts, but not RBC counts in ET patients. In PV patients, JAK2 V617F allele burden was correlated with WBC counts and RBC counts but not PLT counts.

The JAK2 V617F mutation induces constitutive tyrosine kinase activity, and increases sensitivity to cytokines, resulting in a hyperactivity of erythropoietin receptor (EPOR), thrombopoietin receptor (TPOR), and granulocyte clony stimulating factor receptor (G-CSFR). The enhancement of the signal transduction through these receptors leads to hyperplasia of erythrocytic, megakaryocytic and granulocytic progenitor cells [27]–[28]. Consistent with the previous observations, our results showed that RBC counts, WBC counts were much higher in JAK2 V617F-positive patients than wild-type patients. However, platelet counts did not show significant difference between JAK2 V617F mutated patients and wild-type patients. Thus far, the relationship between blood cell counts and JAK2 V617F mutation remain controversial. Peter J Campbell’s study [20] for 806 ET patients from UK demonstrated that PLT counts were lower in JAK2 mutated patients. Antonioli E’s research [21] of 130 ET patients from Italy showed that WBC count was not relevant to JAK2 mutation. Basquiera AL’s study [22] of 43 ET and 45 PV patients from Argentina showed that WBC count and Hemoglobin were relevant to JAK2 V617F, whereas platelet count not, which was similar to our findings. The discrepancies among studies may partly result from ethnic background or sample size.

In the further study for JAK2 V617F mutation rates among patient groups classified by blood cell counts, we found that JAK2 V617F mutation rate increased progressively with increase of RBC counts, and 96.61% patients were JAK2 V617F mutated in patients with RBC counts exceeding 6.5 (6.0)×10^12^/L, confirming the conclusion that JAK2 V617F occurred in almost all the PV patients [13]. In addition, JAK2 V617F mutation rate increasses progressively with WBC count but was not related to platelet counts. Lastly, when lineage hyperplasia state was taken into consideration, JAK2 mutation rate was the highest in trilineage hyperplasia patients, accounting for 93.26%-positiveness, followed by bilineage hyperplasia patients and lowest in monolineage hyperplasia patients. It is worth noting that the high mutation rate was not only frequently observed in PV patients but also in ET patients, suggesting the necessity of routine test for JAK2 V617F mutation in patients with multilineage hyperplasia.

In accordance with Arellano-Rodrigo E ‘s study [23] for ET patients, PDW and MPV were higher in mutated patients than those in wild-type patients in our study, and we also found that P-LCR was higher in mutant group. Since large platelet represented new and immature platelet, our results were consistant with Panova-Noeva M’s study [29], which showed that JAK2 V617F mutation was significantly associated with the increase of immature platelets. It was suggested that JAK2 V617F may play a role in the platelet production and mobilization. Interestingly, this phenomenon existed not only in ET patients but also in all of patients with non-reactive elevated platelet count. Our results support the hypothesis that JAK2 V617F mutation activated TPOR pathway and led to hyperplasia of megakaryocytic progenitor cells [19].

Consistent with previously published data [30]–[31] that the majority of PV patients expressing >50% mutant allele burden and ET patients expressing <50% mutant allele burden, we found that JAK2 V617F allele burden was much higher in our PV patients[median(P_25_–P_75_): 45.02% (35.12%–54.22%)] than in ET patients [median(P_25–_P_75_): 28.23%(17.77%–41.66%)]. However, our JAK2 V617F mutant allele burden was lower than previously reported, which could be due to the fact that JAK2 V617F was examined in purified granulocytes in those studies, but we detected it in whole peripheral blood and bone marrow samples. Because JAK2 V617F primarily occurred in granulocytes while not in T or B lymphocytes [32], the JAK2 V617F allelic ratio was 15% lower on average in whole blood than in granulocytes [33]. It was reported that JAK2 V617F mutant allele burden in purified granulocytes of peripheral blood was more relative with phenotypes of MPDs, especially in treated patients [33], however, it is unlikely to perform JAK2 V617F detection with purified granulocytes in clinical practice, so our study reflects the practical relevance of JAK2 V617F allele burden with blood cell variation pattern. It was also found that JAK2 V617F allele burden was positively correlated with WBC and RBC count, which was consistent with Vannucchi AM’s study [34], in which homozygous patients had higher WBC counts and Hct, regardless of exact diagnosis of ET and PV. It could be attributed to JAK2 V617F dosage-dependent activation of Stat5, Akt, and Erk signaling pathways [35]. Of note, in ET patients, JAK2 V617F showed positive correlation with WBC counts and PLT counts, but not RBC counts, in contrast, it showed positive correlation with WBC counts and RBC counts, but not PLT counts in PV patients. These results suggest that JAK2 V617F mutation may promote granulopoiesis in either ET or PV patients, as previously reported [36]. Considering the fact that ET patients had low JAK2 V617F allele burden while PV patients had high mutant allele burden, we postulate that when the mutant allele burden was low, megakaryopoiesis was enhanced with the increase of mutant allele burden, but erythropoiesis was weak, not increased with mutant allele burden, and when the mutant allele burden was high, the phenomenon reversed. Meanwhile, this inference was also confirmed by BM hyperplasia state analysis.

In conclusion, JAK2 V617F mutation occurs frequently in BCR-ABL negative patients with non-reactive elevated platelet counts. JAK2 V617F mutation should be routinely examined in this population of patients, as recent studies demonstrated that patients with the JAK2 V617F mutation are more susceptible to thrombosis and vascular events [36]–[38]. Furthermore, JAK2 V617F may contribute to increase of RBC counts, WBC counts, P-LCR, PDW, MPV, and lineage hyperplasia, whereas being unrelated with platelet counts. JAK2 V617F mutant allele burden may affect the blood cell proliferation pattern.
